# Supplementary material for: Effects of mechanical weeding on soil fertility and microbial community structure in star anise (Illicium verum Hook.f.) plantations
Source: PLoS One. 2022 Apr 12;17(4):e0266949. doi: 10.1371/journal.pone.0266949 (PMC9004745; doi:10.1371/journal.pone.0266949)
Supplement: S1 Table — (DOCX) [file pone.0266949.s001.docx]

**S1 Table.** **Proportion of Dominant Soil Bacterial Communities at the Genus Level in Star Anise Plantations between the NW and MW Treatments (%).**

| Genus | NW | MW |
| --- | --- | --- |
| *Acidothermus* | 7.79 | 10.26 |
| *norank_f__Xanthobacteraceae* | 6.45 | 8.44 |
| *norank_f__norank_o__Elsterales* | 5.99 | 6.13 |
| *norank_f__norank_o__Subgroup_*2 | 5.39 | 4.61 |
| *norank_f__norank_o__Acidobacteriales* | 3.76 | 3.49 |
| *norank_f__norank_o__norank_c__AD*3 | 4.02 | 3.19 |
| *Bradyrhizobium* | 2.97 | 3.48 |
| *Acidibacter* | 2.76 | 3.10 |
| *Candidatus_Solibacter* | 2.41 | 3.18 |
| *Bryobacter* | 2.06 | 2.53 |
| *unclassified_f__Ktedonobacteraceae* | 2.35 | 2.04 |
| *norank_f__JG*30*-KF-AS*9 | 2.03 | 2.35 |
| *norank_f__norank_o__norank_c__norank_p__WPS-*2 | 2.08 | 2.02 |
| *Burkholderia-Caballeronia-Paraburkholderia* | 2.53 | 1.54 |
| *norank_f__Gemmataceae* | 2.04 | 1.61 |
| *FCPS*473 | 1.64 | 1.96 |
| *Conexibacter* | 1.61 | 1.44 |
| *norank_f__norank_o__IMCC*26256 | 1.37 | 1.72 |
| *unclassified_f__Acetobacteraceae* | 1.46 | 1.55 |
| *norank_f__norank_o__norank_c__TK*10 | 1.28 | 1.62 |
| *norank_f__norank_o__norank_c__Actinobacteria* | 1.29 | 1.41 |
| *Mycobacterium* | 1.27 | 1.30 |
| *norank_f__norank_o__norank_c__Subgroup_*6 | 1.51 | - |
| *Pajaroellobacter* | 1.11 | 1.25 |
| 1921-2 | 1.32 | - |
| *norank_f__norank_o__B*12*-WMSP*1 | 1.06 | - |
| others | 30.45 | 27.34 |

*Note.* NW: no weeding in the star anise plantation, and MW: mechanical weeding in the star anise plantation.
