# Supplementary material for: Effects of mechanical weeding on soil fertility and microbial community structure in star anise (Illicium verum Hook.f.) plantations
Source: PLoS One. 2022 Apr 12;17(4):e0266949. doi: 10.1371/journal.pone.0266949 (PMC9004745; doi:10.1371/journal.pone.0266949)
Supplement: S2 Table — (DOCX) [file pone.0266949.s002.docx]

**S2 Table.** **Proportion of Dominant Fungal Communities at the Genus Level in Star Anise Plantations between the NW and MW Treatments (%).**

| Genus | NW | MW |
| --- | --- | --- |
| *unclassified_k__Fungi* | 22.44 | 20.80 |
| *unclassified_p__Ascomycota* | 9.04 | 23.81 |
| *Saitozyma* | 14.55 | 9.28 |
| *unclassified_c__Agaricomycetes* | 2.03 | 19.18 |
| *Archaeorhizomyces* | 14.14 | 1.48 |
| *Apiotrichum* | 7.06 | 4.64 |
| *unclassified_o__GS*11 | 3.81 | 5.62 |
| *Penicillium* | 4.56 | 2.04 |
| *Trichoderma* | 2.18 | 3.29 |
| *unclassified_f__Clavicipitaceae* | 2.33 | — |
| *Mortierella* | 1.18 | — |
| others | 16.68 | 8.90 |

*Note.* NW: no weeding in the star anise plantation, and MW: mechanical weeding in the star anise plantation.
