# Supplementary material for: Effects of mechanical weeding on soil fertility and microbial community structure in star anise (Illicium verum Hook.f.) plantations
Source: PLoS One. 2022 Apr 12;17(4):e0266949. doi: 10.1371/journal.pone.0266949 (PMC9004745; doi:10.1371/journal.pone.0266949)
Supplement: Attachment — Submitted filename: Response to Reviewers.doc [file pone.0266949.s003.doc]

Dear Editors and Reviewers:

Thank you for your letter and the reviewers’ comments concerning our manuscript entitled “Effects of mechanical weeding on soil fertility and microbial community structure in star anise (*Illicium* *verum* Hook.f.) plantations” (PONE-D-21-29819). Those comments are all valuable and very helpful for revising and improving our paper, as well as the important guiding significance tour researches. We have read comments carefully and have made correction which we hope meet with approval. Revised portion are marked in **Green** in the paper. The main corrections in the paper and the responds to the reviewer’s comments are as flowing:

Responds to the Editors’ and Reviewers’ comments:

Journal Requirements:

When submitting your revision, we need you to address these additional requirements.

1. Please ensure that your manuscript meets PLOS ONE's style requirements, including those for file naming. The PLOS ONE style templates can be found at

https://journals.plos.org/plosone/s/file?id=wjVg/PLOSOne_formatting_sample_main_body.pdf and https://journals.plos.org/plosone/s/file?id=ba62/PLOSOne_formatting_sample_title_authors_affiliations.pdf

***Reply:***  The authors have revised the manuscript style according to the PLOS ONE style templates.

2. In your Methods section, please provide additional information regarding the permits you obtained for the work. Please ensure you have included the full name of the authority that approved the field site access and, if no permits were required, a brief statement explaining why.

***Reply:***  No permits were required. Because the experimental site belongs to the national forest farm, and it is one of the experimental sites of Guangxi Forestry Research Institute.

3. Thank you for stating the following financial disclosure:

“This work was supported by Open Research Fund of Guangxi Key Laboratory of Special Non-wood Forest Cultivation & Utilization (19-B-02-01), Project of key R & D of Guangxi (No. AB1850014) and the eighth batch of special funds for specially invited experts in Guangxi Province, China.”

Please state what role the funders took in the study. If the funders had no role, please state: "The funders had no role in study design, data collection and analysis, decision to publish, or preparation of the manuscript."

If this statement is not correct you must amend it as needed.

Please include this amended Role of Funder statement in your cover letter; we will change the online submission form on your behalf.

***Reply:***  This work was mainly supported by Open Research Fund of Guangxi Key Laboratory of Special Non-wood Forest Cultivation & Utilization (19-B-02-01). However, the money in this fund was not wholly enough to carry out the whole experiment. Therefore, the authors used part of the money in project 2 and 3 for conducting the experiment continuously. The funders had no role in study design, data collection and analysis, decision to publish, or preparation of the manuscript.

4. PLOS requires an ORCID iD for the corresponding author in Editorial Manager on papers submitted after December 6th, 2016. Please ensure that you have an ORCID iD and that it is validated in Editorial Manager. To do this, go to ‘Update my Information’ (in the upper left-hand corner of the main menu), and click on the Fetch/Validate link next to the ORCID field. This will take you to the ORCID site and allow you to create a new iD or authenticate a pre-existing iD in Editorial Manager. Please see the following video for instructions on linking an ORCID iD to your Editorial Manager account: https://www.youtube.com/watch?v=_xcclfuvtxQ

***Reply:***  The corresponding author have linked an ORCID iD to his Editorial Manager account.

Additional Editor Comments:

There appears to be an overall lack of rigor in the experimental setup and data analysis performed. In particular, the authors must include more detailed statistical analysis and additional analyses performed to support the conclusions made.

There appears to be an overall lack of rigor in the experimental setup and data analysis performed. In particular, the authors must include more detailed statistical analysis and additional analyses performed to support the conclusions made.

***Reply:*** The authors have revised the content in manuscript according to the Editor comments.

[Note: HTML markup is below. Please do not edit.]

Reviewers' comments:

Reviewer's Responses to Questions

Comments to the Author

1. Is the manuscript technically sound, and do the data support the conclusions?

The manuscript must describe a technically sound piece of scientific research with data that supports the conclusions. Experiments must have been conducted rigorously, with appropriate controls, replication, and sample sizes. The conclusions must be drawn appropriately based on the data presented.

Reviewer #1: Partly

Reviewer #2: Partly

***Reply:***  The authors have revised this part in manuscript according to the Reviewers’ comments.

2. Has the statistical analysis been performed appropriately and rigorously?

Reviewer #1: No

Reviewer #2: No

***Reply:***  The authors have revised this part in manuscript according to the Reviewers’ comments.

3. Have the authors made all data underlying the findings in their manuscript fully available?

The PLOS Data policy requires authors to make all data underlying the findings described in their manuscript fully available without restriction, with rare exception (please refer to the Data Availability Statement in the manuscript PDF file). The data should be provided as part of the manuscript or its supporting information, or deposited to a public repository. For example, in addition to summary statistics, the data points behind means, medians and variance measures should be available. If there are restrictions on publicly sharing data—e.g. participant privacy or use of data from a third party—those must be specified.

Reviewer #1: Yes

Reviewer #2: No

4. Is the manuscript presented in an intelligible fashion and written in standard English?

PLOS ONE does not copyedit accepted manuscripts, so the language in submitted articles must be clear, correct, and unambiguous. Any typographical or grammatical errors should be corrected at revision, so please note any specific errors here.

Reviewer #1: Yes

Reviewer #2: Yes

5. Review Comments to the Author

Please use the space provided to explain your answers to the questions above. You may also include additional comments for the author, including concerns about dual publication, research ethics, or publication ethics. (Please upload your review as an attachment if it exceeds 20,000 characters)

**Reviewer #1:**

The manuscript is well organized and written but there are some major concerns that need to be addressed in the manuscript before publishing:

Line 40: the authors claimed that "soil microbial community structures are not significantly altered by mechanical weeding", this was claimed in the abstract and the discussion (lines 325, 341)? However, after reading the manuscript I didn’t find any statistical analysis that supports this claim “significantly altered”?? It was also mentioned throughout the manuscript (lines 206, 234, 252, 269, 328, 348, 350) that mechanical weeding altered the microbial diversity of the soil, yet the final conclusion claimed that these reported changes in the microbial diversity were not “significant”… authors need to support and explain this conclusion with statistical analysis.

***Reply:***  The authors have revised this part in manuscript according to the Reviewers’ comments.

The experiment included three replicate blocks (lines 108-112) and that was good for proper statistical analysis for the soil biological properties, yet in the soil microbial diversity analysis the replicates were pooled (line 152) and thus no statistical analysis was done to compare the two groups (no weeding and mechanical weeding) throughout the manuscript.

***Reply:***  The authors have revised this part in manuscript according to the Reviewers’ comments.

Line 163: The authors mentioned that they used “the free online Majorbio Cloud Platform”? The authors should mention in details which tools in the mentioned platform were used for the microbiome analysis… was it QIIME or Mothur or what??? They should also mention which database was used for taxonomy picking and alignment…. was it Greengages or SILVA or what?? they should also mention the version used in the analysis.

***Reply:***  The authors have revised this part in manuscript according to the Reviewers comments.

Figures 1, 2 and 3: the tool/software used to generate these figures should be mentioned in the legend.

***Reply:***  The authors had revised the points where Reviewers suggested in the manuscript. And, the tool/software were also used to generate the figures mentioned in manuscript.

Tables 3 and 4: These tables represent the same data in figure 2, they may move to supplementary materials to avoid redundancy in the manuscript.

***Reply:***  The authors had revised the points where Reviewers suggested in the manuscript.

Line 251: The results showed that two fungal genera were unique in the NW (no weeding ) soil, these two genera didn’t appear in the MW (mechanical weeding) soil, yet throughout the manuscript the authors didn’t rationalize this finding or discussed the functionality of these two genera (are they plant growth-promoting fungi or toxic fungi?? )…. this need to be addressed

***Reply:***  The authors have revised this content in manuscript according to the Reviewers’ comments.

For better contrasting the two soil groups (NW and MW) the authors might check the core microbial community in each group and see whether it is different or not… many free tools are available for this analysis (LEfSe, QIIME.)

***Reply:***  The authors have revised this content according to the Reviewers suggestions. Also, the authors analyzed the microbial communities of the two soil treatments by the LEfSe tool.

**Special thanks to you for your good comments.**

**Reviewer #2:**

In the manuscript by Xiao et al., the authors measure several properties of soil fertility (the activities of three extracellular enzymes and microbial biomass C, N and P) and examine bacterial and fungal taxa in agricultural fields of star anise that were subjected to mechanical weeding. These same soil properties were measured in control plots that were not weeded and compared between the two treatments. The authors conclude that the soil microbial community structure is not altered by mechanical weeding; however, the lack of statistical analysis on the bacterial and fungal community data make such a conclusion impossible, in my opinion. Further, the authors conclude that mechanical weeding improve soil health, but I don’t think the phosphatase and microbial biomass P data support this conclusion. My comments are detailed below. But, overall, I suggest a more rigorous investigation of the data, especially the microbial community data.

***Reply:***  The authors have revised this content in manuscript according to the Reviewers’ comments.

Overall concerns:

The conclusions made about bacterial and fungal community changes are based on proportional representation of different phyla or genera. But there are no statistics conducted on these numbers, despite the authors concluding that no significant differences were found at the phylum level (lines 40 and 326). The authors also make conclusions about genera that change between their treatments (line 347), again without statistics being done on the data. There are programs available that can run t-tests between treatments with corrections for multiple comparisons to test for differences between specific taxa (see STAMP for example https://doi.org/10.1007/978-1-4614-6418-1_780-1). But, what I suggest for this data set is that the authors explore the metagenomic data further before making conclusions, such as by calculating alpha diversity (richness, Shannon diversity, etc.) and beta diversity (i.e., turnover). These will help with whether or not you can conclude that the MW treatment led to “less changed” communities than the NW treatment (lines 349-350). Community data like what are presented here are also typically analyzed with multivariate statistics, such as ordination (principle coordinates analysis, nonmetric multidimensional scaling, etc.) and permutational multivariate analysis of variance (PERMANOVA). Fungal taxa can be assigned to different guilds using FUNGuild (Nguyen et al. 2016, doi: 10.1016/j.funeco.2015.06.006) or the newly available FungalTraits (Polme et al. 2020, doi: 10.1007/s13225-020-00466-2). Exploring how the different fungal guilds changed between treatments could be useful, especially because the Discussion mentions beneficial plant symbionts (i.e., mycorrhizal fungi) (lines 308-311).

***Reply:***  The authors have revised these points according to Reviewers’ suggestion in manuscript. That is, using the FUNGuild and the LEfSe tools in manuscript analyzed the functional predictions of the microbial community.

The phosphatase activity and microbial biomass P data presented here suggest an increase in P demand in the MW plots. The increase in phosphatase activity and decrease in microbial biomass P with weeding, to me, suggests that the organisms were actively acquiring P from organic sources to a greater extent, potentially because inorganic P availability was lower with weeding. Overall, it was difficult for me to follow the conclusions the authors make based on the extracellular enzyme activity and microbial biomass C, N and P data because they don’t compare their numbers with other experiments. What have other studies found with mechanical weeding and how it affects extracellular enzyme activity and assimilated nutrients into microbial biomass? In particular, the difference in microbial biomass C between NW and MW plots reported here (Table 2) is quite large. Is this typical for mechanical weeding? Placing the numbers reported here into context by relating them to other published studies is needed.

***Reply:***  The authors have revised this content in manuscript according to the Reviewers’ comments.

Minor comments:

Line 67: Do weeds really compete with agricultural plants for oxygen? I skimmed reference 12 and don’t see anything about weeds competing with crops for oxygen.

***Reply:***  The authors have revised this content in manuscript according to the Reviewers’ comments.

Line 110: Was each plot divided in half - one side weeded one side not weeded? A methods figure could help here with a diagram of the set up.

***Reply:*** The authors have revised this content in manuscript according to the Reviewers’ comments.

Line 115: Each species of what? Aren't all plant species star anise? Please clarify in the methods.

***Reply:***  The authors re-checked this part and revised part of the content in manuscript according to the Reviewers’ comments.

Lines 162-164: It seems to me that Majorbio is just a cloud for processing data. What pipeline or process was used for the sequence reads? More details are needed on how chimeras were removed, how low-quality reads were filtered out, at what percent OTUs were clustered, etc. If chimeras and low-quality reads were not removed, the conclusions for microbial taxa could be based on sequence errors. The process for analyzing sequence reads needs to be explained in the methods.

***Reply:***  The authors re-checked this part and revised the content in manuscript according to the Reviewers’ comments.

Lines 300-301: I don't follow the conclusion that weeding is not completely harmful to soil fertility. The phosphatase activity and microbial biomass P data suggest increased P demand (i.e., lower P availability) with mechanical weeding. Wouldn’t this be a harmful effect of weeding? P is an essential nutrient to plants. More context is needed on how the enzyme and microbial biomass data compare to other agricultural studies.

***Reply:***  The authors re-checked this part and revised part of the content in manuscript according to the Reviewers’ comments.

Line 307: Fungi are decomposers in more ecosystems than just forests. Please rephrase.

***Reply:***  The authors re-checked this part and revised part of the content in manuscript according to the Reviewers’ comments.

**Special thanks to you for your good comments.**

6. PLOS authors have the option to publish the peer review history of their article (what does this mean?). If published, this will include your full peer review and any attached files.

If you choose “no”, your identity will remain anonymous but your review may still be made public.

Do you want your identity to be public for this peer review? For information about this choice, including consent withdrawal, please see our Privacy Policy.

Reviewer #1: Yes: Mariam Hassan

Reviewer #2: No

**Special thanks to you for your good comments.**

We tried our best to improve the manuscript and made some changes will not influence the content and framework of the paper. We appreciate for Editors/Reviewers’ warm work earnestly, and hope that the correction will meet with approval.

Once again, thank you very much for your comments and suggestions.

Correspondence should be addressed to Prof.& Dr. Shang-dong Yang at the following address, phone number, and email address.

Corresponding author: 1) Name: Wenhui Liang; E-mail address: 723746615@qq.com; Tel: 86-771-2319966; 2) Name: Shangdong Yang; E-mail address: 924433816@qq.com; Tel: 86-771-3270813.

Thanks very much for your attention and consideration. We are looking forward to hearing from you soon.

With kind personal regards,

Sincerely yours,

Jian Xiao (E-mail: 1318513279@qq.com)
