# Supplementary material for: Effects of mechanical weeding on soil fertility and microbial community structure in star anise (Illicium verum Hook.f.) plantations
Source: PLoS One. 2022 Apr 12;17(4):e0266949. doi: 10.1371/journal.pone.0266949 (PMC9004745; doi:10.1371/journal.pone.0266949)
Supplement: Attachment — Submitted filename: Response to Reviewers.doc [file pone.0266949.s004.doc]

Dear Editors and Reviewers:

Thank you for your letter and the reviewers’ comments concerning our manuscript entitled “Effects of mechanical weeding on soil fertility and microbial community structure in star anise (*Illicium* *verum* Hook.f.) plantations” (PONE-D-21-29819). Those comments are all valuable and very helpful for revising and improving our paper, as well as the important guiding significance tour researches. We have read comments carefully and have made correction which we hope meet with approval. Revised portion are marked in **Green** in the paper. The main corrections in the paper and the responds to the reviewer’s comments are as flowing:

Responds to the Editors’ and Reviewers’ comments:

**Additional Editor Comments:**

At this point, the authors have still not included appropriate statistics to support their conclusions despite several suggestions by the reviewers, and so the majority of their conclusions are not supported with appropriate statistics.

***Reply:*** The authors have revised the content in manuscript according to the Editor comments.

**Reviewers' comments:**

**Reviewer #1:**

Regarding the statistical analysis, throughout the manuscript the authors didn’t mention the used tests to analyse the data. As a reader and reviewer we need to know the used statistical test and wether it was appropriate or not. Wherever you mention any significance with P value you need to mention the used statistical test.

***Reply:***  The authors have revised the content in manuscript according to the Reviewer comments.

**Special thanks to you for your good comments.**

**Reviewer #2:**

While I think the data collected by the authors for this manuscript are valuable, the study still lacks proper statistical analysis for the conclusions that are being made. I do see that both FUNGuild and LEfSe were used to analyze the data, but a large portion of the Results and Conclusions are still based on changes in the abundances of different taxa and no statistics are conducted on these numbers.

***Reply:***  LEfSe is a software for discovering high-dimensional biomarkers and revealing genomic features to distinguish between two or more biological conditions (or taxa). The software first uses the non-parametric factorial Kruskal-Wallis (KW) sum-rank test (non-parametric factorial Kruskal-Wallis rank sum test) to detect features with significant abundance differences and to find taxa that differ significantly from the abundance. Finally, LEfSe used linear discriminant analysis (LDA) to estimate the magnitude of the effect of each component (species) abundance on the difference effect. Therefore, we can directly identify bacteria and fungi with significant differences in NW and MW by LEfSe directly, without the need to do a separate test for differences between groups.

In addition, we had used R language (version 3.3.1) to perform Wilcoxon rank-sum test and graphs, compared taxonomic identities and functional groups from FUNGuild of NW and MW.

Lines 235-302: These results and the conclusions that follow are all just observations on how the abundance of different taxa change. Statistics need to be conducted to make conclusions about how the taxa changed. As I suggested previously, I recommend using the free software STAMP (https://doi.org/10.1007/978-1-4614-6418-1_780-1). The website is here: https://beikolab.cs.dal.ca/software/STAMP. This program will allow you to conduct t-tests between the MW and NW treatments. Both taxonomic identities and functional groups from FUNGuild can be compared (lines 341-348).

***Reply:***  We have used R language (version 3.3.1) to perform Wilcoxon rank-sum test and graphs, compared taxonomic identities and functional groups from FUNGuild of NW and MW.

Lines 305-307: There are plenty of statistics that do analyze community data at the OTU level. As I suggested previously, community data like what are presented here are also typically analyzed with multivariate statistics, such as ordination (principle coordinates analysis, nonmetric multidimensional scaling, etc.) and permutational multivariate analysis of variance (PERMANOVA). These statistics can all be run in the vegan package of the program R. You can download R for free here: https://www.r-project.org/.

***Reply:***  We have added Principal Component Analysis (PCA) to analyze community data at the OTU level. And R language (version 3.3.1) for PCA statistical analysis and graphing also was used.

Discussion: I previously commented "The phosphatase activity and microbial biomass P data suggest increased P demand (i.e., lower P availability) with mechanical weeding. Wouldn’t this be a harmful effect of weeding? P is an essential nutrient to plants. More context is needed on how the enzyme and microbial biomass data compare to other agricultural studies." In the revised manuscript, I don't see any inclusion of extracellular enzyme production or microbial biomass nutrient content from other agricultural studies. This will help put the data presented in the current study into context. in addition, I do not see any discussion of changes to P availability with mechanical weeding. Given the increase in phosphatase production and decrease in microbial biomass P with mechanical weeding, I think that there is lower P availability with mechanical weeding. The way I interpret the data presented, the mechanical weeding increases assimilation of nutrients by the soil microbes (thus the increase in organic C and organic N content). But, organic P content does not show this same pattern. Instead, phosphatase activity increases with mechanical weeding. This means that there is not enough inorganic P available, so the microbes need to put their energy into releasing phosphatase to free up P that is bound in organic matter. In other words, the data suggest that there is a higher P demand or lower P availability with mechanical weeding. I understand that the authors may disagree with me here, but the Discussion overall lacks these kinds of conclusions and mostly just re-states the results.

***Reply:***  Some types of weeds have good phosphorus uptake capacity, which can accumulate soil surplus phosphorus in biomass and promote phosphorus biocycling (Roy 2017). However, without total topsoil inversion, the plant materials cut by a brush cutter and incorporation into the soil which provide fertility is limited. A stratified layer of phosphorus builds up on or near the soil surface, increasing the risk of phosphorus release and subsequent transport of dissolved reactive phosphorus via surface runoff and tile drain water (Ulén et al, 2010). We speculate that this may be the reason why microbial biomass phosphorus was significantly lower in the MW treatment than in the NW treatment.

Roy ED. Phosphorus recovery and recycling with ecological engineering: A review. Ecol Eng. 2017;98: 213-227.

Ulén B, Aronsson H, Bechmann M, Krogstad T, ØYgarden L, Stenberg M. Soil tillage methods to control phosphorus loss and potential side-effects: a Scandinavian review. Soil Use Manage. 2010; 26(2): 94-107.

**Special thanks to you for your good comments.**

We tried our best to improve the manuscript and made some changes will not influence the content and framework of the paper. We appreciate for Editors/Reviewers’ warm work earnestly, and hope that the correction will meet with approval.

Once again, thank you very much for your comments and suggestions.

Correspondence should be addressed to Prof.& Dr. Shang-dong Yang at the following address, phone number, and email address.

Corresponding author: 1) Name: Wenhui Liang; E-mail address: 723746615@qq.com; Tel: 86-771-2319966; 2) Name: Shangdong Yang; E-mail address: 924433816@qq.com; Tel: 86-771-3270813.

Thanks very much for your attention and consideration. We are looking forward to hearing from you soon.

With kind personal regards,

Sincerely yours,

Jian Xiao (E-mail: 1318513279@qq.com)
